# Effect of apricot kernel seed extract on biophysical properties of chitosan film for packaging applications

**DOI:** 10.1038/s41598-024-53397-2

**Published:** 2024-02-10

**Authors:** Mona Saied, Azza Ward, Shimaa Farag Hamieda

**Affiliations:** Microwave Physics and Dielectrics Department, National Research Centre, Cairo, Egypt

**Keywords:** Biophysics, Environmental sciences

## Abstract

Chitosan is a natural biodegradable biopolymer that has drawbacks in mechanical and antibacterial properties, limiting its usage in biological and medicinal fields. Chitosan is combined with other naturally occurring substances possessing biological antibacterial qualities in order to broaden its application. Ethanolic apricot kernel seed extract was prepared, analyzed, and incorporated into chitosan film with different concentrations (0.25, 0.5, and 0.75 wt%). Furthermore, the effect of AKSE and γ-radiation (20 Gy and 20 kGy) on the physical properties of the film was studied. The prepared films were characterized by Fourier transform infrared spectroscopy (FTIR), which revealed that AKSE did not cause any change in the molecular structure, whereas the γ-irradiation dose caused a decrease in the peak intensity of all concentrations except 0.75 wt%, which was the most resistant. In addition, their dielectric, optical, and antimicrobial properties were studied. Also, AKSE-enhanced optical qualities, allowed them to fully block light transmission at wavelengths of 450–600 nm. The dielectric properties, i.e., permittivity (ε′), dielectric loss (ε′′), and electrical conductivity (σ), increased with increasing AKSE concentration and film irradiation. The antimicrobial studies revealed that the antimicrobial activity against *Escherichia coli* and *Canodida albicans* increased with AKSE incorporation.

## Introduction

The improvement of life quality over the past few decades has resulted in a constant rise in the global population, which has resulted in an excessive consumption of resources and, as a result, a sizeable creation of waste. Therefore, excessive production may be caused by ineffective waste management. This procedure demands enormous expenses and may harm both the ecology and the health of living things. In order to reduce the amount of the produced waste and associated socioeconomic costs, it is vital to adopt a recycling and reuse mindset^1^. Fruits and vegetables are among the most consumed foods worldwide. Biowaste, including fruit and vegetable peels and seeds, is generated in enormous amounts, raising environmental pollution^2^. Fruit and vegetable by-product management recently converted from a linear economy to a more sustainable circular one. This could reduce the overuse of raw resources and enable the waste to be used for its potential economic and biological worth^3^. The best way to minimize this waste is to use them in the food, pharmaceutical, and associated industries before or after the proper extraction of bioactive compounds from them. Agricultural wastes are used to prepare extracts and manufacture polymeric films that can be used in industrial and medical applications, solving pollution problems and providing national income. Due to their structures and qualities, peels and seeds are waste products that can be used in medical and pharmaceutical applications. These products may supply antioxidant substances, such as phenols and flavonoids, which have greater antioxidant activity. In recent years, there is an increase in the use of natural extracts or their original components, such as low molecular weight phenolic acids, tannins, and flavonoids, for food packaging applications^4^. Due to growing environmental consciousness and worries about the use of renewable natural resources, a lot of work has been put into developing eco-friendly and biodegradable materials for the creation of the next generation of composite products. Natural materials are now once again popular due to ecological concerns. When considering a more sustainable future, recycling and environmental safety are taking on greater implication^5–7^. Biomaterials are becoming increasingly important in the creation of a sustainable environment. Despite the recent discovery of biomaterials for drug delivery, tissue engineering, and medical diagnostics, there has been significant advancement in both physical and chemical techniques for controlling biological reactions^8^. Chitosan, a cationic polysaccharide, is a deacetylated derivative of chitin, a biopolymer that is mostly derived from marine sources and is widely distributed and renewable^9,10^. Due to its notable qualities, including non-toxicity, biodegradability, biocompatibility, inherent antibacterial activity, and good film-forming ability, chitosan has been thoroughly researched for prospective uses in the biomedical, biotechnological, pharmaceutical, and food sectors^11–13^. The creation of chitosan-based films and coatings, which have become a successful and environmentally benign method to increase the shelf life of perishable goods, is one of the most promising uses of chitosan^14^. Chitosan-based films could be a sustainable substitute for synthetic plastics that are unsafe for the environment^15,16^. After the integration of several components that can improve the antioxidant and antibacterial activities and hence the protective capacity, they can also be employed as active food packaging^17,18^. Numerous plant extracts have been investigated so far with regard to how they affect the technological and functional properties of packaging films meant to preserve food^19^. In some of the most recent investigations, chitosan-based films that contain plant extracts such as *Camelina sativa* seed oil (*Pistacia terebinthus*) stem, leaf, and seed extracts have been examined^20^, *Berberis crataegina* fruit extract and seed oil^21^, *Prunus armeniaca* kernel essential oil^22^, *Eucalyptus globulus* essential oil^23^, and tea extracts^24,25^. Apricot fruits are a favorite for consumption. Their kernels, however, are also a plentiful source of fascinating nutrients. However, the kernels are frequently left unutilized during the preparation of apricots. A variety of antioxidants with antibacterial qualities can be found in their extracts. Chitosan films, including different concentrations of AKEO, were prepared using the solvent casting method and used for active food packaging applications for the very first time. Fourier transform-infrared FT-IR spectra and field emission scanning electron microscope FE-SEM images confirmed its successful incorporation into the film. The films also exhibit brilliant antimicrobial and antioxidant properties as compared to neat chitosan films and successfully prevent the fungal growth on packaged bread slices^22^. The most widely utilized synthetic, petroleum-based, non-biodegradable polymers for the creation of active packaging materials are traditional. After consumption, the packaging materials ultimately contribute to the non-biodegradable municipal solid waste that tends to build and pose major environmental risks. This opens the door for the use of natural biodegradable polymers in active food packaging^26^. The dielectric characteristics of a material can offer useful information regarding the mobility of polymers' chemical chains that contain dipolar regions or dipolar side groups^27^. The movement of ions in a separate second phase, which is primarily made up of water and glycerol, is what primarily causes the conductivity to increase as the glycerol content is increased in potato starch films^28^. The domains of bound and free water in the film were attributed, respectively, to a constant value and a linear region in the dielectric permittivity of CS films, which showed a considerable dependence on the moisture content^29^. The electrochemical properties of sodium alginate polymer film combined with *Withania somnifera* leaf extract (WLISA) made by the solution casting method were explored in the current inquiry, which deals with alternating current electrical properties^30^. The effect of different doses (10, 20, and 30 kGy) of gamma irradiation on biocomposites based on PLA, rosemary extract, and PEG (20 wt%) was evaluated. It was found that the modification in structure, morphology, and thermal properties of the materials depends on the irradiation dose and the presence of the natural extract^31^.

The main objective of our study is to enhance the physical, antioxidant, and antimicrobial properties of chitosan films incorporated with apricot kernel seed extract improving, their resistance to sterilizing gamma radiation doses for packaging applications.

## Experimental

### Materials and methods

#### Materials

Chitosan, molecular weight 100,000–300,000 was purchased from Molekulargewicht, New Jersey USA. Glutraldehyde, 25% solution, density: 1.09 g/ml, BIO BASAIC CANADA INC and apricot from local markets.

##### Preparation of the extract

Apricot seeds were collected, dried, powdered, and combined for 24 h with 70% ethanol. A vacuum rotary evaporator was used to filter and concentrate the resultant ethanol extracts^32^.

##### Determination of total phenolic content

The Folin-Ciocalteu method was used to determine the total phenolic content^33^. In a nut shell, the extract (100 µL) was put into a test tube, the volume was adjusted to 3.5 mL with distilled water, and 250 µL of Folin–Ciocalteau reagent was added to oxidize the extract. After 5 min, 1.25 mL of a 20% aqueous sodium carbonate (Na_2_CO_3_) solution was added to the mixture to neutralize it. The absorbance was measured at 725 nm against a solvent blank after 40 min. Using a calibration curve made using gallic acid, the total phenolic content was calculated and represented as milligrams of gallic acid equivalent (mg GAE) per 100 g of sample.

##### Determination of radical ABTS scavenging activity

According to^34^, the stock solutions of the ABTS* reagent were made by combining 2.45 mM potassium persulfate and 7 mM ABTS* in equal parts and allowing them to react for 16 h at room temperature (25 °C) in the dark. Following that, the working solution was created by combining 1 mL of ABTS* solution with 60 mL of ethanol and water (50:50, v/v) to get an absorbance of 1.0 ± 0.02 units at 734 nm. In a dark environment, 4.95 mL of the ABTS* solution was added to the extracts (50 µL) and left to react for 1 h and measured at 734 nm. The following equation was used to get the percent inhibition of the ABTS* free radical:

$${\text{Inhibition }}\left( \% \right) = {1}00 \times \left[ {\left( {{\text{A}}_{{{\text{control}}}} - {\text{A}}_{{{\text{sample}}}} } \right)/{\text{A}}_{{{\text{control}}}} } \right]$$

where A_control_ is the absorbance of the control reaction (containing all reagents except the test compound) and A_sample_ is the absorbance with the test compound.

##### Preparation of chitosan/AKSE films

Chitosan was dissolved in distilled water to obtain a concentration of 1 wt%. Different concentrations of AKSE were incorporated into the solution to obtain concentrations of (0, 0.25, 0.5, and 0.75 wt%). The prepared films were crosslinked with 2 ml of a 25% glutaraldehyde solution. The solutions were then poured into Teflon molds, and they were dried in an oven at 45 °C for 20–24 h^35^.

##### Radiation facility

A Canadian gamma cell-40 (137Cs) was used for the irradiation at the NCRRT in Nasr City, Cairo, Egypt. Samples underwent a single dose level of 20 kGy γ-ray exposure.

##### Attenuated total reflection-Fourier transform infrared spectroscopy (ATR-FTIR)

Using the JASCO FT/IR 300 E (Tokyo, Japan), the functional groups in the composite films' backbones were located and recorded. 400–4000 cm^−1^ ATR-FTIR spectroscopy experiments.

##### Scanning electron microscope (SEM)

Scanning electron microscopy was used to analyze the morphology of the polymer blends' surfaces, bulk, and structural alterations. The scanning electron microscope (SEM) Philips XL30 Japan model with the energy dispersive spectroscopy (EDX) equipment, which is installed at the National Research Centre (NRC), was used to create the electron micrographs.

##### Dielectric measurements

Using a high-resolution broadband impedance analyzer (Schlumberger Solartron 1260), the samples' conductivity and dielectric characteristics were examined. The applied AC electric field had a frequency range of 0.1 Hz to 1 MHz. Through the use of a GPIB cable (IEE488), the impedance analyzer was connected to a personal computer to automate the measurements and calculations. Data was collected using the commercial interfacing and automation program Lab VIEW. Additional details are available elsewhere. The measurements' error percentages of ε′ and tan δ are 1% and 3%, respectively. A temperature regulator with a Pt 100 sensor was used to regulate the samples' temperature. There is a 0.5 °C inaccuracy in the temperature measurements^36^.

##### Thermo gravimetric analysis TGA

The Perkin-Elmer, TGA 7 (USA) instrument was used to test the samples' thermal stability. Up to 700 °C, the temperature was raised at a rate of 10 °C/min in a nitrogen environment.

##### Optical properties using ultraviolet–visible spectroscopy

Every film was sliced into a rectangle sample measuring 4.5 × 0.5 cm, which was then placed in a quartz cell and put into a Cintra 303 UV–Vis spectrophotometer (made by GBC Scientific Equipment, located in Mexico City). The reference utilized was air. Each spectra was collected at wavelengths between 200 and 800 nm, and each film was put to the test three times. Transmittance percentage (%T) averages were used to report the results^37^.

##### Antibacterial and antifungal assays

*Preparation of inoculum* Gram-positive bacteria (*Staphylococcus aureus*), Gram-negative bacteria (*Escherichia coli*), and pathogenic yeast (*Candida albicans*) were used in this study as the pathogenic microorganisms used for the inoculation, which were made from fresh overnight broth cultures using nutrient broth medium. The inoculum was adjusted so that the plates and flasks got 1.5 × 10^8^ cfu/mL of both types of bacteria and the fungus. The following procedure describes a method for preparing the desired inoculum by comparison with a 0.5 McFarland standard^38^.

#### Methods

Qualitative evaluations were conducted in nutritional broth, according to Barry, A.L.^39^, which was composed of yeast extract, peptone, meat extract, and NaCl at concentrations 2,5,1, and 5 g/L respectively, and incubated at 37 °C^40^. 25.0 µL of both bacterial and fungal suspensions was inoculated separately into each 100.0 mL conical flask containing 25.0 mL of the sterile nutrient broth medium (NB). The prepared chitosan samples were applied to the previously inoculated flasks separately and then incubated in a shaker incubator utilizing the shake-flask technique. For antibacterial activity calculation, the microbial inhibition was determined by the colony-forming unit (CFU) by two main techniques. The first is the plate total viable count technique, which involves inoculating petri dishes containing solidified nutrient agar medium with 100 µL from a (10^–4^) dilution of bacterial and fungal strains and incubating them at 37 °C, while the second is the optical density (O.D.) technique, in which the reduction of both bacterial and fungal cells by chitosan samples were measured by spectrophotometry. In both methods, the reduction growth rate R (%) for treated chitosan samples in comparison to the untreated control samples was observed^41^.

$${\text{Relative }}\left[ {{\text{Reduction }}\left( \% \right)} \right] \, = \left( {{\text{A}} - {\text{ B}}/{\text{A}}} \right) \times { 1}00$$

where B is the number of microorganisms present in tested flasks following the application of the tested treated chitosan samples and, A is the number of microorganisms present in control flasks containing only pathogenic strains without chitosan treatment.

### Ethical approval

All the methods were carried out in accordance with relevant institutional guidelines and regulations.

## Results and discussion

### Determination of total phenolic content and radical ABTS scavenging activity

The amount of phenol in AKSE was estimated through the standard gallic acid calibration curve (y = 0.237 x + 0.0455), and the radical scavenging activity, a method based on the ability of seed extract to reduce ABTS* free radical, was determined as described in the experimental part. The results were calculated from a calibration curve (y = 3.421 x + 0.1877) and expressed in Trolox equivalents (TE) per 100 g. The results of the total phenolic and antioxidant activity, which were represented in Table 1, indicated that the total phenol was 358 ± 0.05 mg GAE/100 g, and the mean value of the antioxidant activity was 410 ± 0.014 mg TE/100 g. Our results indicate the high value of the total phenolic compound and total antioxidant activity of AKSE. These results agreed with the results from another study, which found that the high value of total phenol and the antioxidant activity were measured in apricot with sweet kernel extract (330 ± 174 mg GAE/100 g) and (753 ± 240 mg TE/100 g)^42,43^.

**Table 1:** Total phenol and antioxidant activity of AKSE extract.

ABTS (mg TE/100 g)		Total phenol (mgGAE/100 g)	
Mean	SD	Mean	SD
410	± 0.014	358	± 0.05

The results are expressed as mean ± SD of three experiments.

### Attenuated total reflection-Fourier transform infrared spectroscopy (ATR-FTIR)

**Figure 1:**
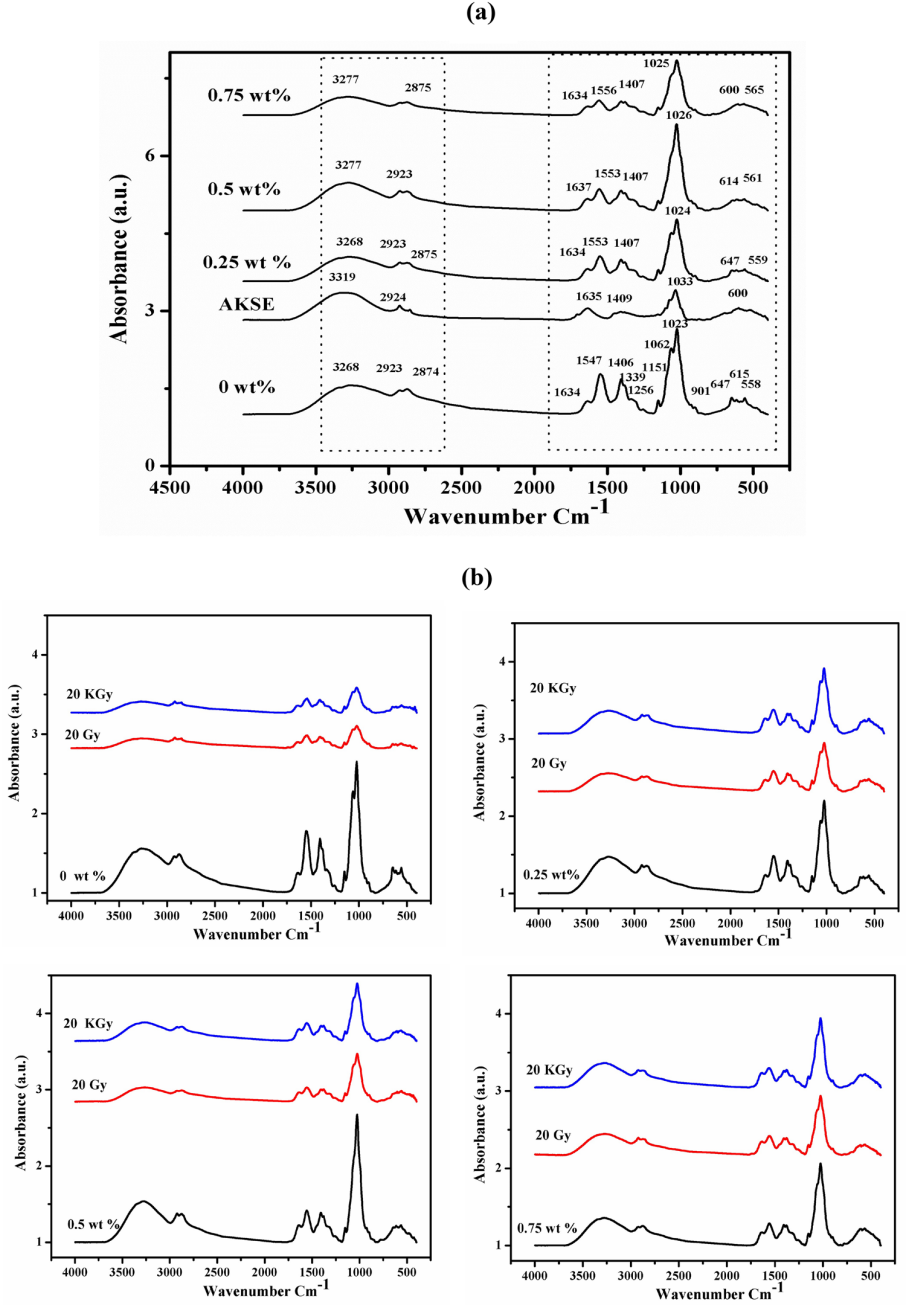
FTIR spectra of Chitosan/AKSE films of concentrations (0, 0.25, 0.5 and 0.75 wt%) (**a**) and for irradiated and unirradiated films with 20 Gy and 20 kGy gamma-ray (**b**).

The interaction between chitosan and AKSE was investigated using FTIR spectroscopy. Figure 1a shows the spectrum of pure chitosan, AKSE, and chitosan/AKSE films at concentrations of 0.25, 0.5, and 0.75 wt% in order to thoroughly characterize the starting materials. The distinctive peaks for the stretching vibration of the hydroxyl OH group were at 3600–3000 cm^−1^ and at 292–2875 cm^−1^ for the symmetric and asymmetric stretching vibrations of the C–H and N–H, respectively. The carbonyl bonds C=O of the amide group CONHR's vibration at 1645 cm^−1^ and the vibrations of the protonated amin group at 1556–1547 cm^−1^ were both related to the absorption at the range of 1680–1480 cm^−1^. Methylene and methyl groups' bending vibrations were discernible at 1339 and 1406 cm^−1^, respectively. CO group vibrations have been implicated in absorption in the range of 1160–1000 cm^−1^^44^. The ring COH, COC, and CH2OH's CO atoms are responsible for the distinctive bands near 1062–1023 cm^−1^. The wiggling of chitosan's saccharide structure is what's responsible for the modest peak at 901 cm^−1^, 610–700 s, and broad ≡C–H bend^45^. Upon loading the AKSE, a sharp peak at 1026 cm^−1^ will occur at a concentration of 0.5 wt% after the two peaks at 1062 and 1023 cm^−1^ overlapped together. Additionally, there is only slight evidence of additional bands emerging following the addition of AKSE. This finding suggests that the active groups of the polymer and the extract do not interact chemically. Only the peak intensity is reduced by a rising AKSE concentration. This indicates a better interaction between the polymer and the extract.

Figure 1b shows the effect of different doses of gamma radiation on Chitosan/AKSE at concentrations of 0, 0.25, 0.5, and 0.75 wt%. It is evident from the spectra that the FTIR-spectra of the irradiated and unirradiated films are comparable, and there are no changes noticed in the functional groups. Only the notable changes in the peaks intensity. From the figures, we noticed that, pure chitosan and the two composite films of concentrations 0.25 and 0.5 wt%, have no chemical changes after irradiation by the two γ-irradiation doses (20 Gy and 20 kGy). Only a high decrease in the peak intensity. Also, we notice that, the two irradiation doses have approximately the same effect. As shown in Fig. 1b, the composite film of concentration of 0.75 wt% affected slightly by gamma radiation exposure. Also, there was no notable changes in the peaks intensity of FTIR spectra of the film. This indicates that, the composite film of concentration 0.75 wt% is the most resistant to the effective γ-radiation doses. Similar results were obtained in a previous study on the effect of frankincense oil on alginate film of concentration 1%, and the FTIR spectra revealed no significant structural changes occurred after exposing to sterilizing dose of γ-radiation between (25–30 kGy)^35^.

### Scanning electron microscope (SEM)

**Figure 2:**
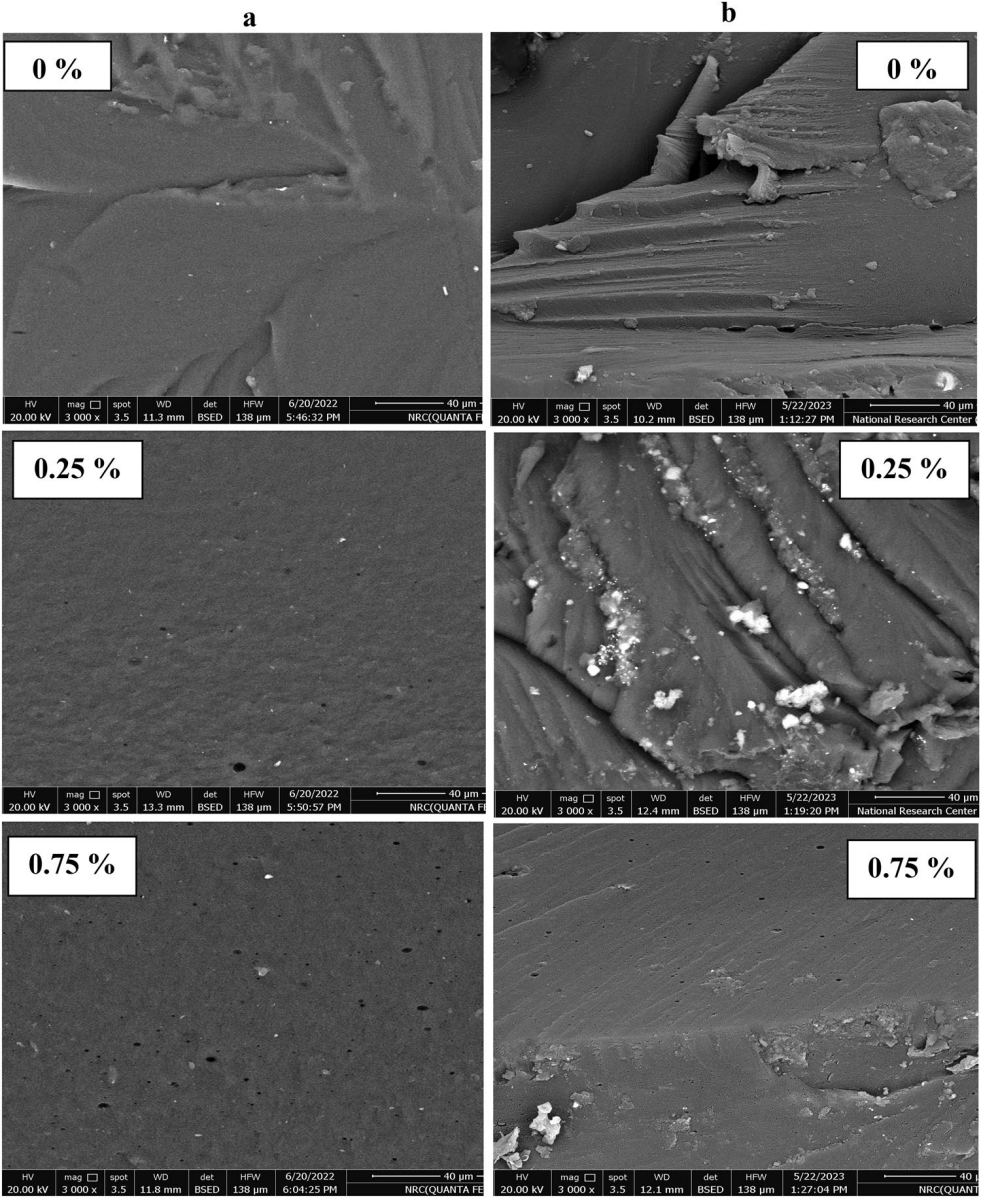
SEM micrographs of (**a**) unirradiated and (**b**) γ-irradiated chitosan/AKSE films of concentrations (0, 0.25, 0.5 and 0.75 wt%) with 20 kGy γ-radiation dose.

The SEM images of γ-irradiated and unirradiated Chitosan/AKSE films cross sections of concentrations 0, 0.25, and 0.75 wt% are shown in Fig. 2a,b respectively. We observed that, in Fig. 2a, pure chitosan film showed smooth and regular aspects. However, a noticeable change in the chitosan film microstructure was distinguished when AKSE was added to the film. Also, no irregularities or breaks were detected, indicating a homogenous distribution of AKSE inside the chitosan polymer matrix. In addition, dark droplets were observed for 0.25 and 0.75 wt% films. This is a result of extract droplets being entangled in the polysaccharide network. As the extract's concentration increased, the droplets grew larger. This might be explained by an increased frequency of collisions between extract droplets, which would likely lead to coalescence. The oil phase in the film is visible in the cross-section photographs, and this may also explain why the film cuts less precisely than pure chitosan film. These oil droplets were not spherical like usual oil or water emulsions. This might be related to the traction forces created on the chitosan network by solvent evaporation^23^. The use of *Citrus limonia* essential oil with chitosan films has been associated with similar behavior^46^. Figure 2b shows the cross-section images of γ-irradiated films with 20 kGy. We noted the presence of some cracks, which indicates that the sample was affected by the radiation dose to which it was exposed, and the cracks decreased by extract addition and almost disappeared in the 0.75 wt% sample. So, we concluded that this concentration is the most resistant when exposed to γ-irradiation. Although it appeared that the doses of 20 kGy did not have a major critical impact due to the high energy of γ-radiation, this suggests that these doses can be used safely to sterilize chitosan/AKSE films.

### Dielectric properties

**Figure 3:**
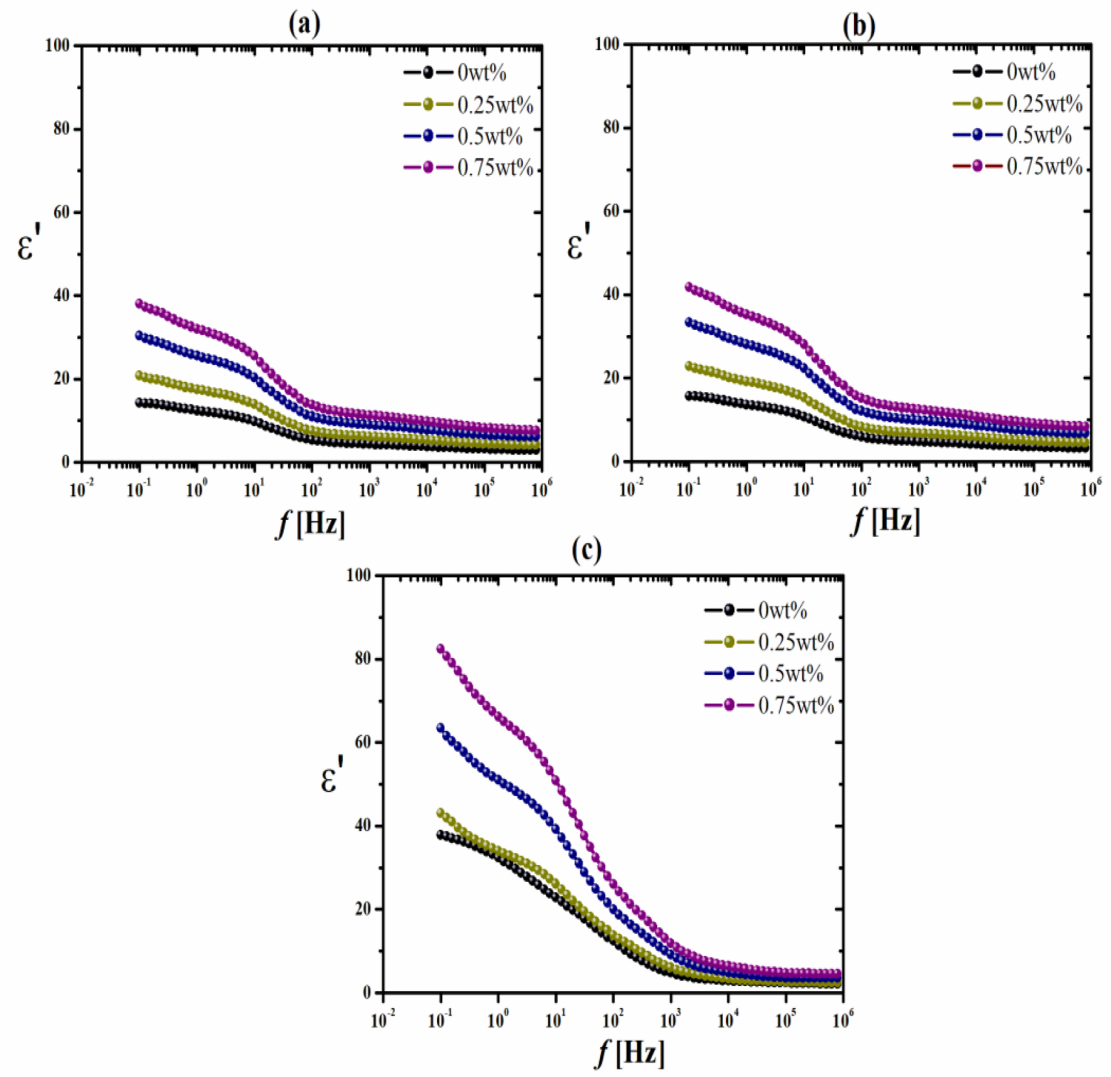
Variation of the permittivity ε′ vs frequency *f* of un-irradiated and γ-irradiated chitosan/AKSE composite films with different doses; (**a**) un-irradiated. (**b**) 20 Gy γ- irradiated. (**c**) 20 kGY γ-irradiated.

Concerning the dielectric characterization of chitosan/AKSE, Fig. 3 illustrates the frequency dependences of the permittivity ε′ for chitosan/extracts films, γ-irradiated chitosan/AKSE, and 20 kGy γ-irradiated chitosan/AKSE films loaded with various amounts of extract at room temperature 30 ± 1 °C. Due to high interfacial polarization and electrode effects, all films exhibit a notable rise in the permittivity ε′ at low frequencies^47^. At frequencies greater than 10 Hz, the material's intrinsic dielectric characteristics become apparent. All dielectric materials exhibit a continual reduction in permittivity ε′ with increasing frequency.

One of the methods to increase ε′ would be by raising the polarization of the material when exposed to an electric field, with the orientational or dipolar mechanism being the ideal option for this use. It is generally known that the polarization mechanism cannot proceed when the frequency of an electric field is heightened. The contribution of polarization to the dielectric constant will decrease as the frequency of the electric field increases because the mechanism of polarization cannot keep up with the change in the electric field. Different polarizations are frequently used to improve the dielectric properties of composite films. For instance, the irregular charge distributions caused by the heterogeneity of composite materials permit space charge polarizations in an electric field. As the radiation dose is increased, ε′ increases due to the deep orientation and accumulation of charges creating the acetate and NH_3_ + ions in Figs. 3a and 5a. Radiation causes chain scission, which may make tiny entanglements more mobile by weakening the interchain connections^35^. However, higher values of ε′ are preferable for antibacterial action. The microbial cell wall is seriously harmed by these NH_3_ + positive ions^48^.

**Figure 4:**
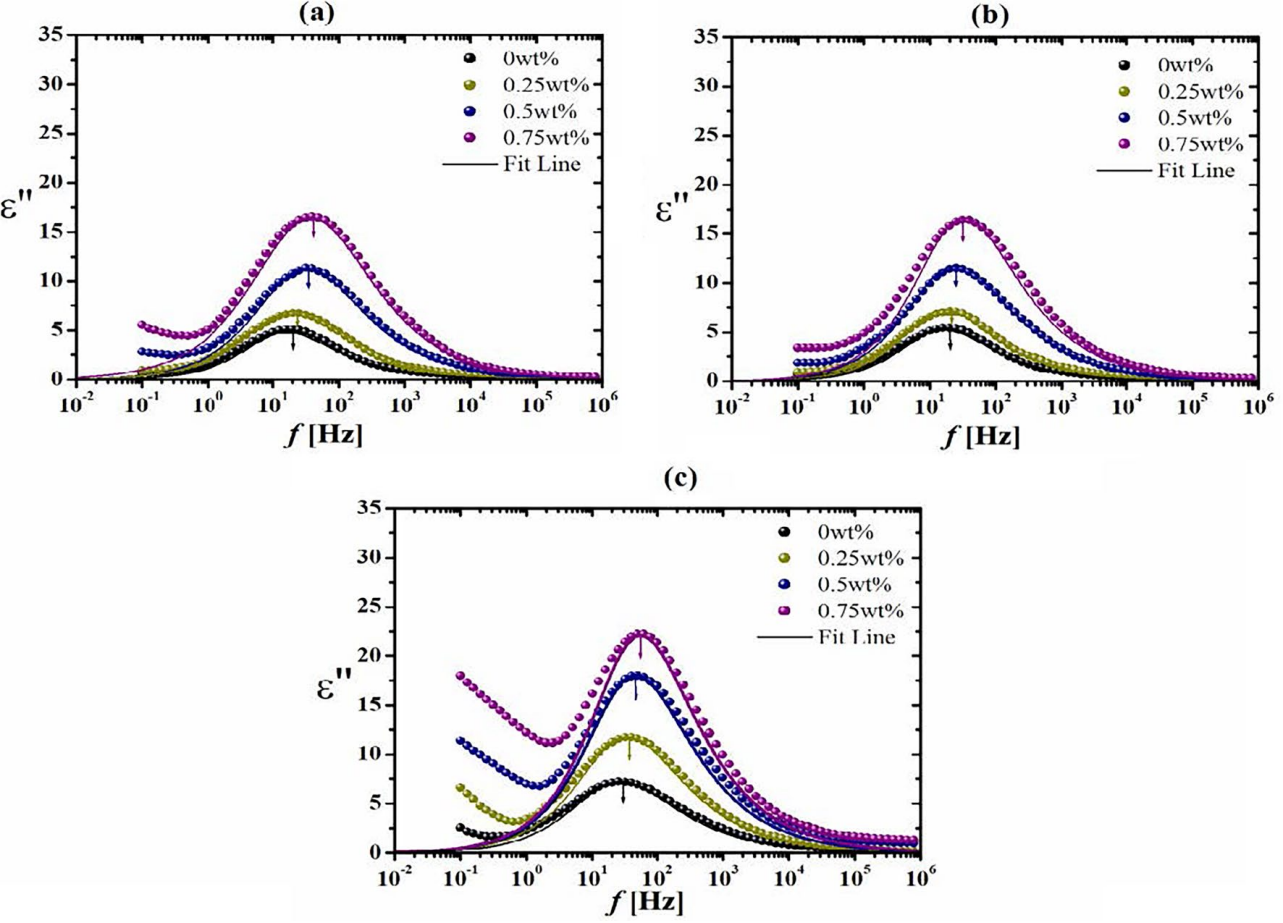
Variation of the dielectric loss ε" vs frequency *f* of un-irradiated and γ-irradiated chitosan/extract composite films with different doses; (**a**) Un-irradiated, (**b**) 20 Gy γ-irradiated. (**c**) 20kGY γ-irradiated. Fit lines in accordance with Havriliak–Negami (HN) functions.

**Figure 5:**
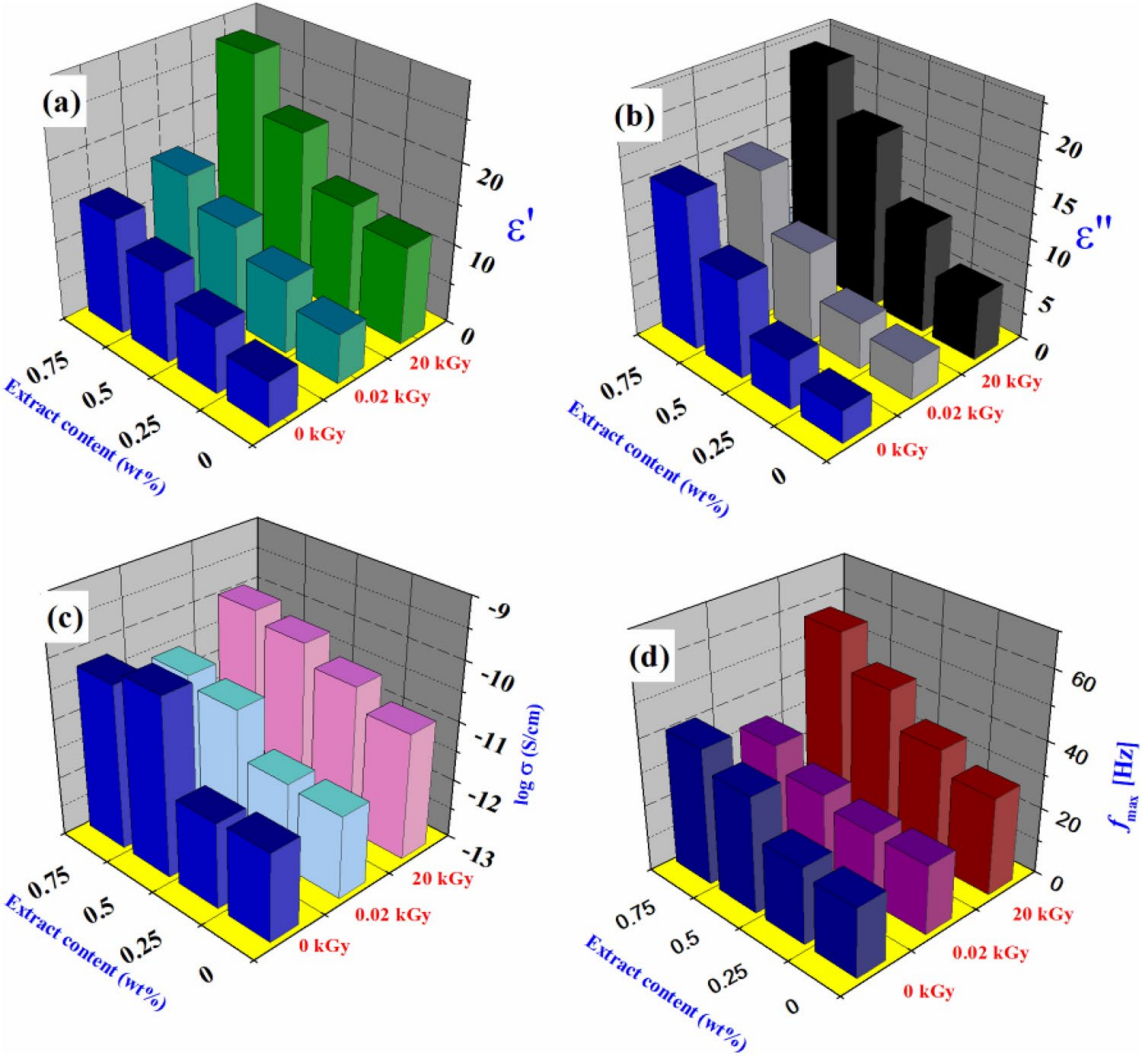
Variation of the permittivity ε′, dielectric loss ε", conductivity σ and maximum frequency* f*_max_ with extract content at different doses of γ-irradiation.

On the other hand, the decrement of ε″ values with frequency existing in Fig. 4 may be due to the build-up of charges at the composite interfaces, referred to Maxwell–Wagner–Sillars effect^49^. Also, it might be due to the development of space charges at a lower frequency. Additionally, Fig. 5b shows the increase in ε′′ values caused by increasing the radiation dose at a constant frequency. This increase is assigned to the enhancement of the conductivity σ of the composites (Fig. 5c) resulting from charge migration in chitosan/AKSE composites. This increase encountered in σ values was experienced as a result of increasing the radiation dose, attributed to high charge carrier motion supported by larger segmental motion of the chitosan backbone brought on by chain scission^50^. This result confirms the dielectric results. However, at low radiation doses (20 Gy), crosslinking occurs between chitosan chains, resulting in an immobile network due to hydrogen bonds and decreased conductivity^51^. The dielectric findings are supported by this result. However, at modest radiation doses (20 Gy), chitosan chains crosslink, leading to a stationary network due to hydrogen bonds and a drop in conductivity.

Returning to Fig. 4, the plot of ε″ vs. frequency revealed the presence of a main peak with an exponential decrease in response to frequency. The height of this peak increased with increasing extract content and irradiation doses. The ε″ data in Fig. 4 can be represented in terms of Havriliak–Negami functions^35^. The fitting process showed two relaxation processes. The main relaxation at lower frequencies is related to the rotational / segmental motion of charge carriers, whereas the other relaxation at higher frequencies is ascribed to the motion of the polar side group^52^.

The variation of the maximum frequency* f*_max_ of the lower frequency peak obtained from the fitting with extract content at different doses of γ-irradiation is shown in Fig. 5d. It is seen that *f*_max_ increased with increasing doses of γ-irradiation due to the rise in the number of charge carriers and free radicals in the host matrix^53^. However, no significant changes are encountered in the maximum frequency* f*_max_ of the higher frequency relaxation for all composites before and after irradiation.

### Thermo gravimetric analysis (TGA)

**Figure 6:**
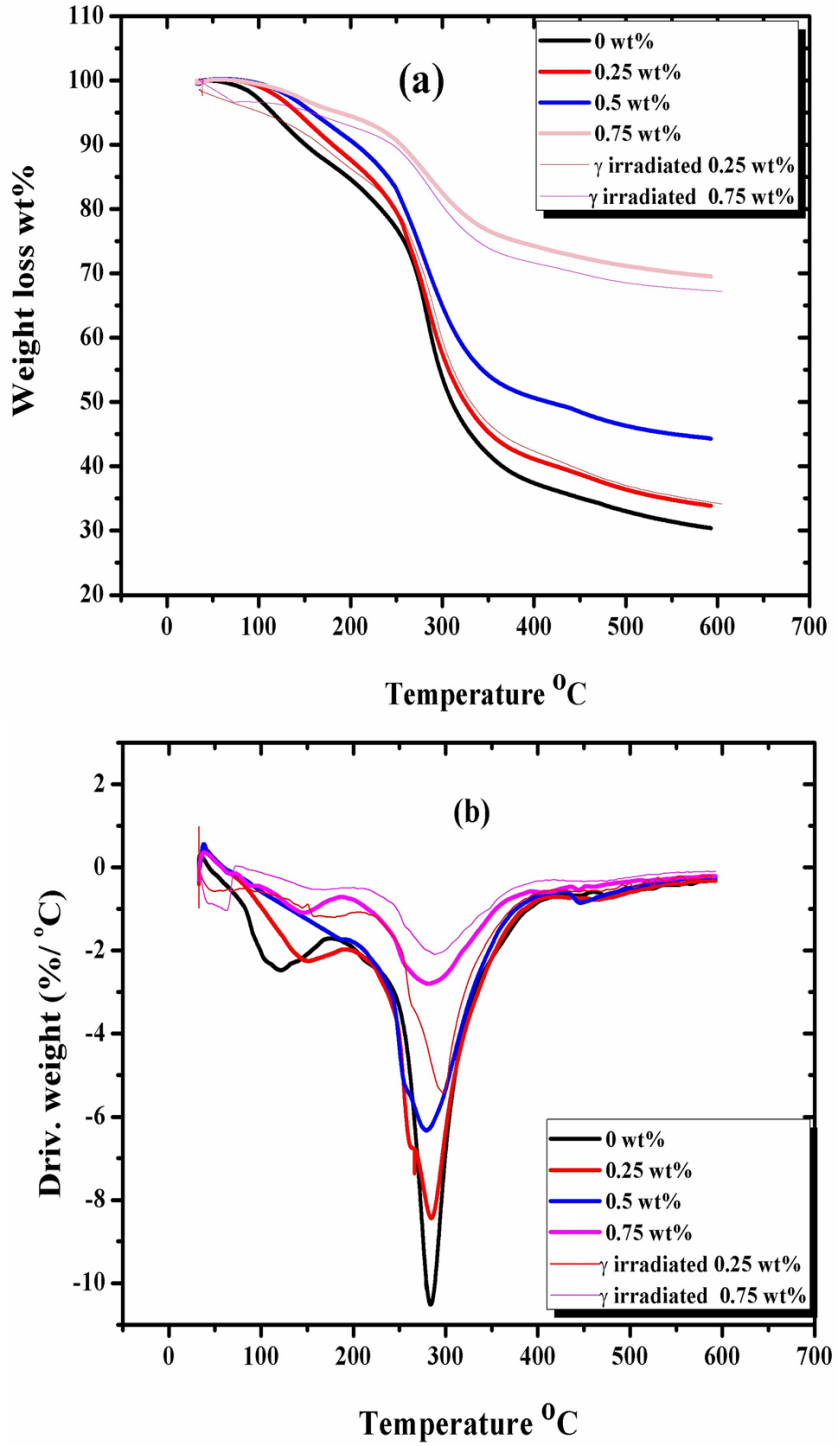
TGA and DTG curves curve of unirradiated chitosan/AKSE of concentrations (0, 0.25, 0.5 and 0.75 wt%) and γ-irradiated (0.25 and 0.75 wt%) films.

The mass of a sample in response to temperature or the length of time it was exposed to thermal breakdown can be calculated by thermogravimetric analysis (TGA). The effect of AKSE on chitosan film mass loss was studied and illustrated in Fig. 6. There is an interesting observation that chitosan and its films at concentrations of 0, 0.25, 0.5, and 0.75 wt% have two degradation steps. The analytical method counts the mass of materials that vary as they go through oxidation, reduction, decomposition, or evaporation^54^. Table 2 displays a detailed summary variance of the examined samples' 10, 25, 50, and 70 wt% weight reduction wt%. From the data, it is clear that, in the initial degradation step of 10 wt% weight loss, chitosan is faster than its films, which were incorporated with kernel extract. The onset temperature (T_o_)_,_ at a loss mass of 10 wt%, increased as the concentration of the extract increased. Therefore, it is a good result that kernel seed extract addition increases the stability of chitosan film. From the figure, we also notice that weight losses of 25, 50, and 70 wt% shift to higher values for the films containing the extract than the control, and that the concentration of 0.75 wt% is the most stable film^55^. For AKSE loaded into chitosan films, the DTG curves in Fig. 5b show a single decomposition peak for chitosan films only, but after AKSE incorporation, two decomposition peaks with shoulders are found. Their mass losses have a slight tendency to increase after γ-irradiation. The chitosan crosslinking likely also played a role during irradiation^31^. According to Fig. 6b, the sample containing 0.75 wt% AKSE appears to be more stable to radiation than the sample containing 0.25 wt%. From the data illustrated in Table 2, we deduce that the residual weight loss (∆w) values generally increase with the increased AKSE concentration and radiation dose as, was also found in other studies^56^.

**Table 2:** TGA data of chitosan/AKSE of concentrations (0, 0.25, 0.5 and 0.75 wt%) and irradiated (0.25 and 0.75 wt%) films.

Sample concentration	Temp. at characteristic weight loss (°C)				Residual mass %
	10 wt%	25 wt%	50 wt%	70 wt%	
0 wt%	150	255	309	588	30
0.25 wt%	182	261	325	0	33.7
0.5 wt%	208	274	412	0	44.57
0.75 wt%	254	381	0	0	67.23
Irradiated 0.25 wt%	167	267	331	0	34.39
Irradiated 0.75 wt%	245	338	0	0	69

### Optical properties

**Figure 7:**
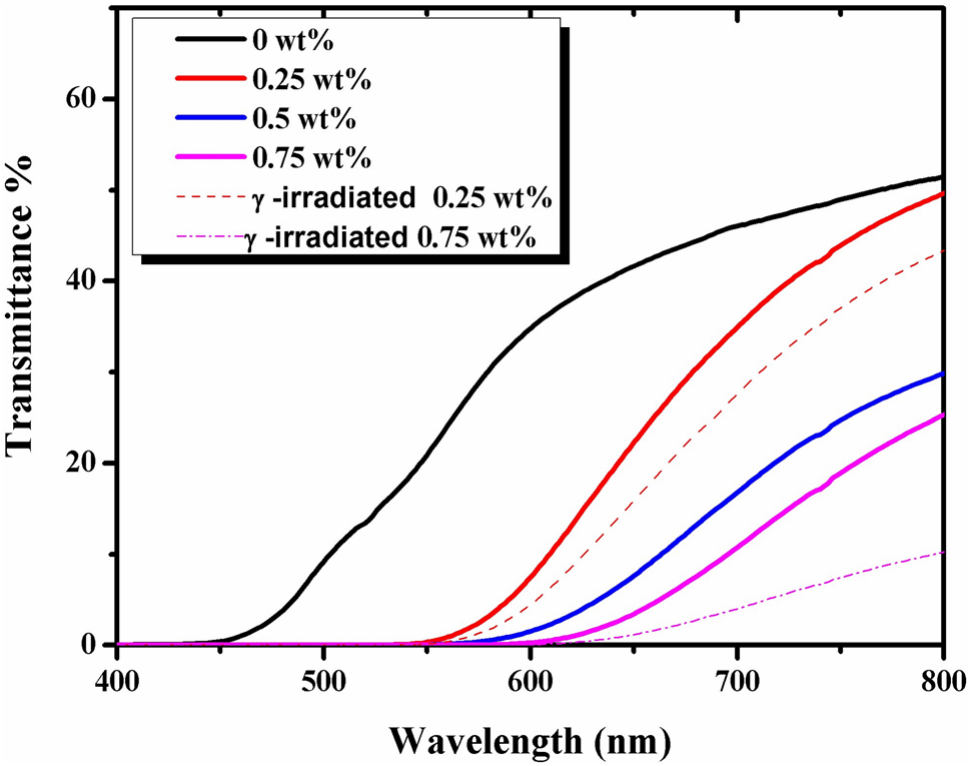
The UV/Vis. Transmittance % spectra of unirradiated chitosan/AKSE of concentrations (0, 0.25, 0.5 and 0.75 wt%) and γ-irradiated (0.25 and 0.75 wt%).

The ability of packaging material to act as a barrier against the energy in the UV–vis light region is an added value in the context of the photostability of specific food compounds. Therefore, the optical properties of chitosan-based films were studied in terms of the light transmittance in the UV–vis light region, and the results are presented in Fig. 7. Transmittance values in the UV range (≤ 450 nm) for chitosan and chitosan/AKSE films were 0%. A complete blockage of the UV light at wavelengths up to ∼ 450 nm may be due to the UV light absorption arising from the presence of crosslinking components. While the transmittance values in the UV range (≥ 450 nm) for chitosan films range from 0 to 52% and for chitosan/AKSE concentrations of 0.25, 0.5, and 0.75 wt% films were from 0 to (50, 30, and 25%) respectively. The maximal transmittance (evaluated at the wavelengths 800 nm) in 0.75 wt% film was reduced by ∼ 27 percentage points compared to the chitosan films (i.e., from 52 to ∼ 25%). Therefore, The incorporation of AKSE increases the wavelength at which the film could transmit light. The higher the concentration of AKSE, the greater transmittance reduction at the wavelengths 800 nm. This can indicate that the higher the extract content, the higher absorption capacity of the films in the visible light region. The reduction of light transmission could be attributed to polyphenol compounds of AKSE, which improve the n → π^∗^ absorption in the UV region^57^. As the superior light absorption capacity of the film at a concentration of 0.75 wt% is evident, it can be discussed that these films could be further selected for the design and production of packaging materials specifically tailored to block the most damaging light wavelengths^58^. The effect of γ-radiation on chitosan/AKSE films at concentrations 0.25 and 0.75 wt% was also studied and illustrated in Graph 7. The data revealed thatthe transmittance of light decreased with γ-irradiation. This may be due to the effect of γ-radiation energy, which influences its colors, increasing the tone of the film for a stronger yellowish color, increasing the opacity , and decreasing the light transmittance. When a film is meant to be used as food packaging, its opacity becomes extremely important. For items that are susceptible to light-catalyzed degradation reactions, shielding against incident light may be necessary in certain situations^59^. This is a good result that these films are recommended to be used for food and medical packaging applications.

### The antibacterial and antifungal assays

**Figure 8:**
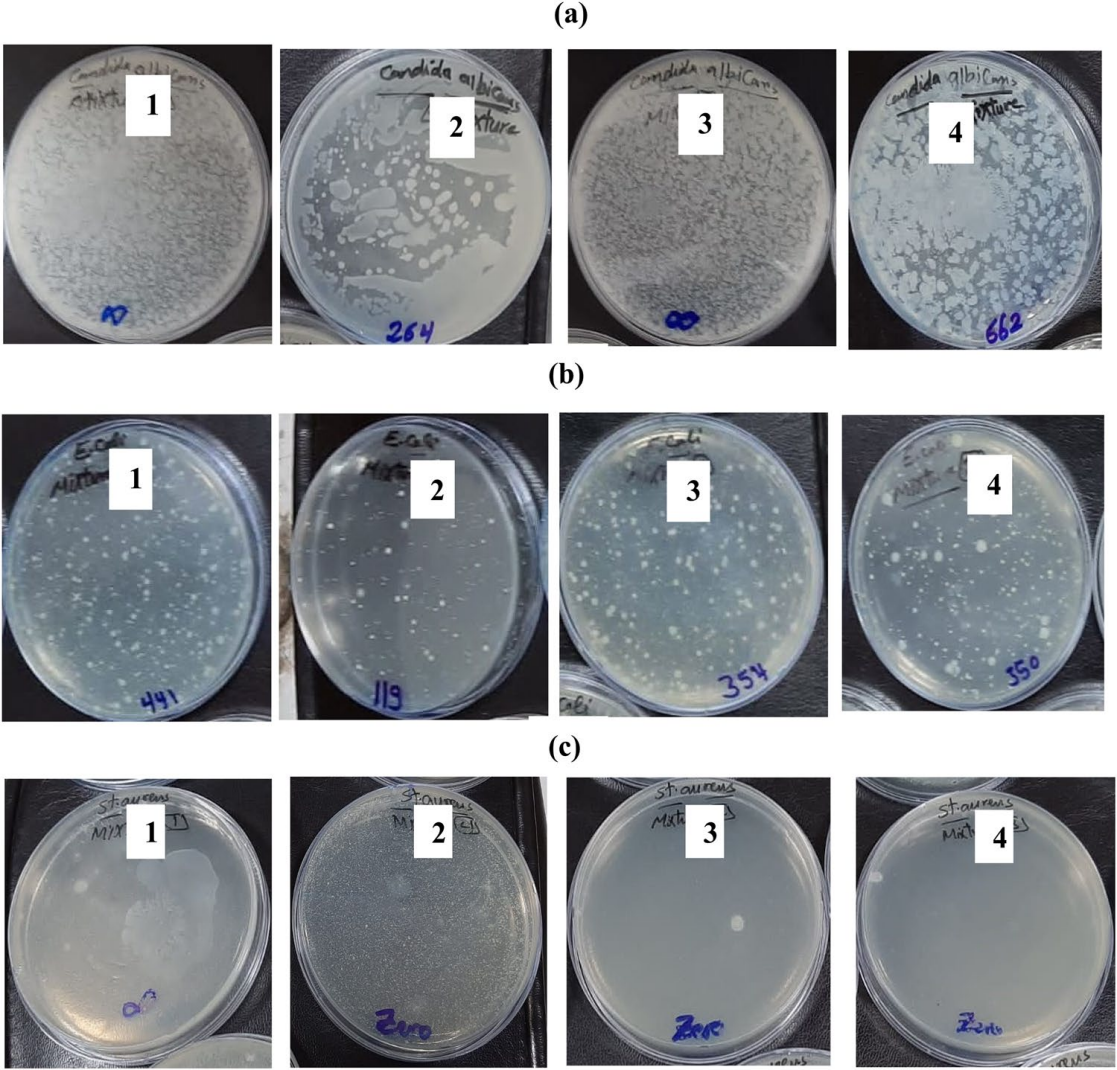
CFU of the tested samples (1) chitosan, (2) AKSE, (3) 0.5 wt% and (4) 0.75 wt% using shake flask method after incubation applying plate total viable count technique by (**a**) Candida albicans, (**b**) Escherichia coli and (**c**) Staphyllococcus aureus.

**Figure 9:**
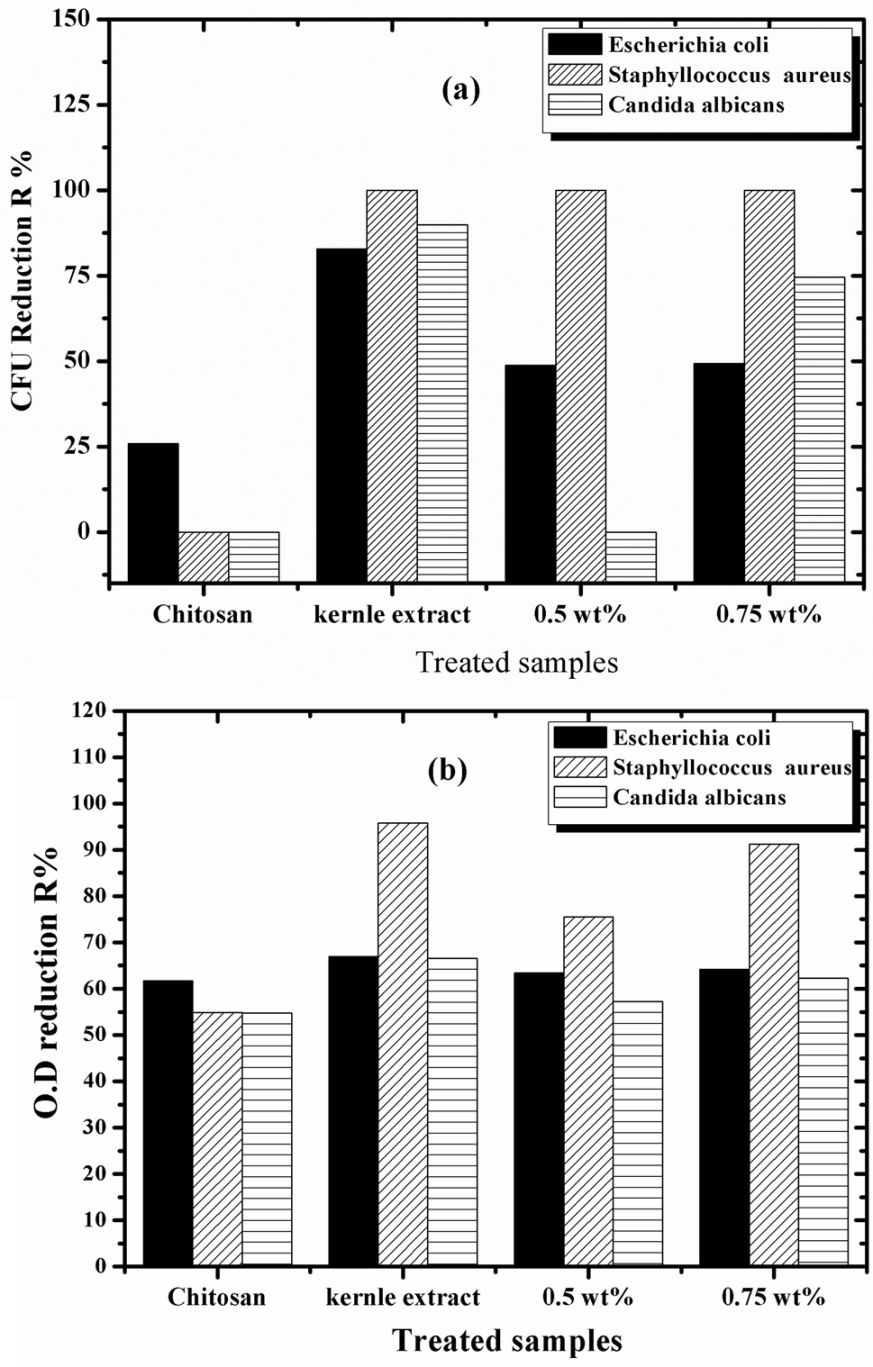
CFU reduction R% of the tested samples chitosan, AKSE, 0.5 wt% and 0.75 wt% applying (**a**) plate total viable count technique and (**b**) optical density (O.D.) technique.

The antimicrobial activity of AKSE, chitosan, and the prepared films at concentrations of 0.25 and 0.75 wt% was studied against *Candida albicans* (ATCC 10231), *Escherichia coli* (ATCC25922), and *Staphylococcus aureus* (ATCC 6538) by CFU and OD reduction%^60^. The results gained by CFU applying plate total viable count technique were illustrated in Table 3 and Figs. 8 and 9a. From the results, it is clear that pure chitosan has no activity against *Staphylococcus aureus* and *Candida albican* but has a CFU reduction % of 0.25% against *Escherichia coli*. However, AKSE has the best antimicrobial activity, reaching 100% against *Staphylococcus aureus*. Upon loading AKSE into the film, we noticed that the antimicrobial activity of the film against *Staphylococcus aureus* was enhanced, and reaching 100%. Further, the effect of AKSE was not obvious against *E. coli* and *C. albicans*. On the other hand, the antimicrobial efficacy was studied for the samples by optical density O.D. The results were illustrated in Table 4 and Fig. 9b. From the results, we noticed that pure chitosan films had the least antimicrobial activity against all microbial species, while AKSE had the highest activity against them^43^. In addition, the O.D. reduction R% increased with AKSE loading into the film, and the film with a concentration of 0.75 wt%had the best antimicrobial activity against all the microbial species, and the highest effect was against *S. aureus*. However, AKSE enhances the antimicrobial efficacy of chitosan films. *S. aureus* produces deep-seated infections, including osteomyelitis and endocarditis, as well as more severe skin infections, in addition to being the main factor in hospital-acquired surgical wound nosocomial infections^61^. The fact that *S. aureus* was the strain that was observed to be the most sensitive to these films is an important finding. All samples also have an excellent effect on *Staphylococci,* which can cause many forms of skin infection.

**Table 3:** CFU reduction R% of the tested samples using shake flask method after incubation applying plate total viable count technique.

Treated samples	CFU reduction R% by test bacteria		
	*Escherichia coli*	*Staphyllococcus aureus*	*Candida albicans*
Chitosan 0 wt%	25.90	Nil	Nil
AKSE	82.78	100	89.85
0.5 wt%	48.77	100	Nil
0.75 wt%	49.35	100	74.54

**Table 4:** CFU of the tested samples using shake flask method after incubation applying optical density (O.D.) techniqu.

Treated sample	Optical density (O.D.) R%		
	*Escherichia coli*	*Staphyllococcus aureus*	*Candida albicans*
Chitosan 0 wt%	61.41	54.9	54.81
AKSE	66.92	95.83	66.59
0.5 wt%	63.73	75.48	57.24
0.75 wt%	64.22	91.21	62.24

## Conclusion

Apricot kernel seed extract was prepared and analyzed for total antioxidant capacity and total phenols. The total phenols were 350 mg GAE/100 g, and the antioxidant capacity was 410 mg TE/100 g. The extract was incorporated into chitosan film with different concentrations (0, 0.25, 0.5 and 0.75 wt%) and its effect on chitosan properties has been studied. Further, the effect of gamma radiation at 20 Gy and 20 kGy on the film was also studied. The prepared films were characterized by Fourier transform infrared spectroscopy (FTIR), scanning electron microscopy (SEM), and thermogravimetric analysis (TGA) in addition to studying their dielectric, optical, and antimicrobial properties. FTIR revealed that AKSE did not cause any change in the molecular structure. Whereas the gamma irradiation dose caused a decrease in the peak intensity of all concentrations except 0.75 wt%, which was the most resistant one. The Pure chitosan film showed smooth and regular aspects. However, a noticeable change in chitosan film microstructure was distinguished when AKSE was added to it. Also, no irregularities or breaks were detected, indicating a homogenous distribution of AKSE inside the chitosan polymer matrix. After irradiation, γ-irradiated films with 20 kGy showed some cracks, indicating that the samples were affected by the radiation dose to which they were exposed, and the cracks decreased by adding extract and almost disappeared in the 0.75 wt% sample. So, we concluded that this concentration is the most resistant when exposed to γ-irradiation.

The dielectric properties, permittivity ε′, dielectric loss ε′′ and electrical conductivity σ, increased with increasing AKSE concentration and irradiation. The ε′ increased due to durable charge orientation and accumulation, creating the acetate and NH_3_ + ions. High-charge carrier motion supported by greater segmental motion of the chitosan backbone brought on by chain scission is responsible for the increase in values observed as a result of increasing radiation exposure. Also, AKSE enhanced optical qualities allowed them to fully block light transmission at wavelengths of (450–600) nm.

The antimicrobial studies revealed that AKSE had better antimicrobial activity against *Escherichia coli* and *Candida albicans* and the antimicrobial activity of the films increased with AKSE incorporation. So, due to the better physical and antimicrobial properties of chitosan/AKSE films, they are recommended to be used for food or medical packaging applications.

## Data Availability

All data generated or analysed during this study are included in this published article.
