# Author Correction: Effect of apricot kernel seed extract on biophysical properties of chitosan film for packaging applications

**DOI:** 10.1038/s41598-024-55674-6

**Published:** 2024-03-04

**Authors:** Mona Saied, Azza Ward, Shimaa Farag Hamieda

**Affiliations:** Microwave Physics and Dielectrics Department, National Research Centre, Cairo, Egypt

Correction to: *Scientific Reports* 10.1038/s41598-024-53397-2, published online 10 February 2024

The Acknowledgements section in the original version of this Article was omitted. It now reads:

“The authors greatly appreciate the National Research Centre due to the financial support provided by Project (No. 13010419).”

The original Article has been corrected.

